# Causal linkage between angiotensin-converting enzyme 2 and risk of lung cancer: a bidirectional two-sample Mendelian randomization study

**DOI:** 10.3389/fmed.2024.1419612

**Published:** 2024-07-08

**Authors:** Shubin Chen, Ruiling Ning, Wei Jiang, Shaozhang Zhou, Qitao Yu, Haijie Gan

**Affiliations:** Medical Oncology of Respiratory, Guangxi Medical University Cancer Hospital, Nanning, China

**Keywords:** angiotensin-converting enzyme 2, lung cancer, causal risk factors, GWAS dataset, Mendelian randomization study

## Abstract

**Background:**

Observational studies suggest a connection between ACE2 (angiotensin-converting enzyme 2) and lung cancer. However, it's not apparent if confounding variables are interfering with the link. Therefore, we aimed to define the relationships between ACE2 and the risk of lung cancer.

**Methods:**

With the aim of developing genetic tools, we selected SNPs substantially associated with ACE2 using a statistically significant criterion. The relevant SNPs were then taken from the lung cancer GWAS dataset for additional research. After that, we used two-sample Mendelian randomization (MR) to ascertain if ACE2 is causally linked to the risk of developing lung cancer. To investigate the causal links' directions, we also performed a reverse MR analysis.

**Results:**

According to our findings, there is strong evidence that ACE2 is linked to a decreased chance of developing lung cancer (odds ratio: 0.94; 95% confidence interval: 0.90–0.98; *P* = 0.0016). The IVW method, the major MR analysis, was not impacted by heterogeneity in any of the analyses, according to Cochrane's Q test ($P_{\text{Cochran}{\text{e}}^{\text{′}}\text{sQ}}$ = 0.207). The MR-Egger intercept (*P*_intercept_ = 0.622) showed no indication of horizontal pleiotropy in any of the investigations. Outlier SNPs were not detected by the MR-PRESSO global test (*P*_globaltest_ = 0.191). The leave-one-out analysis was performed, and the results showed a steady outcome. Nonsignificant causal estimates between lung cancer and ACE2 were produced by reverse MR analysis.

**Conclusion:**

MR investigation revealed a significant causal link between ACE2 and the risk of getting lung cancer. These findings may have implications for public health measures aimed at reducing the incidence of lung cancer.

## Introduction

Lung cancer (LC) is a lethal tumor that threatens human life and health. According to the World Health Organization's most current “Global Cancer Statistics 2020” statistics, lung cancer accounts for more than 1/10 of all malignant tumors worldwide, with 2.2 million new cases and 1.8 million fatalities in 2020 (1). Since symptons do not arise early in non-small cell lung cancer (NSCLC) development, about 62% of patients receive the initial diagnosis at stage IV (2). On top of that, 5-year overall survival of advanced NSCLC patients was only 5% (3). Finding the modifiable protective or risk factors is critical to halting the development of lung cancer.

ACE2 is a critical regulator of the renin-angiotensin system (RAS), which regulates vascular tone and fluid and electrolyte balance (4). The 40 kb long human ACE2 gene is located on chromosome Xp22. The gene has 18 exons, the bulk of which are comparable to those in ACE (5). Like ACE, ACE2 possesses a zinc metalloprotease domain at its N-terminus that is visible outside the cell. In terms of structure, ACE has two enzymatically active sites, whereas ACE2 has just 1. ACEIs, or angiotensin-converting enzyme inhibitors, are potent antihypertensive medications (6). Despite evidence that these medications are short-term safe, worries have been raised that long-term usage may be linked to a higher risk of cancer (7–9). Many recent studies have found that the use of ACEIs increases the risk of lung cancer, indicating that ACE may be a preventive factor against lung cancer (10–12). The heart, respiratory system, intestines, kidneys, and pancreas are a few of the tissues known to have ACE2 (13). By operating on the ACE/Ang-II/AT1R axis, ACE2 modulates RAS and several pathological processes, including fibrosis, hypertension, cardiac dysfunction, and acute respiratory distress syndrome (ARDS) (14, 15). The alveolar and bronchiolar epithelium, the endothelium, and the smooth muscle cells of the pulmonary arteries of rats all express ACE2, whereas bronchiolar smooth muscle cells do not (16). The protective effect of ACE2 against acute lung damage has been demonstrated in some animal studies (17–19), although it is still unclear if ACE2 acts as a preventative factor for lung cancer. *In vitro*, lung cancer cell growth has been shown to be inhibited by overexpressing ACE2 in two prior studies (20, 21), suggesting that this may have a protective effect. However, the results of these two studies may have been tainted by the introduction of potential confounding, and there is a lack of additional evidence to investigate the causal relationship between them.

MR is a technique for evaluating the causal relationship between risk variables and illness. As instrumental variables (IVs), genetic variations are used in MR to successfully avoid the effects of confounding factors, which are challenging to control in observational studies (22). When compared to natural randomized controlled trials, which can assess the causal relationship between exposure and outcome at the genetic level while excluding the possibility of reverse causality (23), MR analysis is similar because the alleles affecting genetic variants are randomly assigned to offspring at conception and are unaffected by environment and other unknown confounding factors.

To fully describe the relationships between ACE2 and the risk of lung cancer, we conducted a two-sample MR analysis in this investigation using summary data from genome-wide association studies (GWAS). This study added to the body of research supporting the significance of ACE2 as a modifiable risk factor for lung cancer.

## Materials and methods

### Study assumption

The STROBE-MR statement, which was just created and is used to report MR research, was followed in our study (24). Three key presumptions form the foundation of MR: The following three points apply to IVs: (1) ACE2 and IVs have an especially strong connection; (2) IVs shouldn't be associated with known or unknowable confounding variables; and (3) IVs only influence lung cancer through ACE2 (25). The two-sample MR approach was used to investigate the potential causal relationship between ACE2 and lung cancer. SNPs, or single nucleotide polymorphisms, are variations in a single nucleotide at the genomic level that result in DNA sequence polymorphisms. It is brought on by the conversion, subversion, insertion, or deletion of a single base. Known as a single nucleotide, it is frequently found in both human and animal genomes. It is also the most common of the heritable human variants. In MR studies, SNPs are used as IVs.

### Genetic instruments for ACE2

The dataset ebi-a-GCST90010102 was chosen for our search of the angiotensin-converting enzyme 2 levels reported in the IEU Open GWAS project (https://gwas.mrcieu.ac.uk/). This dataset, which encompassed 1,301 samples and a total of 18,166,693 SNPs with the primary population being Europeans, was taken from a study by the authors Gilly A (26) that was published in 2020. We identified SNPs strongly related with ACE2 to create genetic tools using a statistically significant criterion (P < 1 × 10^−5^, linkage disequilibrium (LD) r^2^ < 0.001, LD distance > 10,000 kb).

### GWAS data on lung cancer

GWAS summary data of lung cancer was extracted from the International Lung Cancer Consortium (ILCCO) (27). This dataset covered data from 27,209 patients with a total of 8,945,893 SNPs and the main population is also European. SNPs highly correlated with ACE2 were extracted by the above method, and then the corresponding SNPs were extracted in the lung cancer GWAS dataset for further analysis.

### Preprocessing of SNPs data

The SNPs screened by the above methods may also have the possibility of inconsistent and unmatched effect alleles, We used RStudio to harmonize to solve this problem. To ensure that SNPs were strong instrumental variables, we performed F statistics. The formula of F statistics is F = R^2^ × (N-2)/(1 – R^2^), where N represents the sample size and R^2^ refers to the variance of ACE2 levels explained by IVs. Only the SNPs with F statistics >10 were considered to be included in the MR analysis. To satisfy the third critical hypothesis, the SNPs only affect lung cancer through ACE2, SNPs cannot be strongly associated with lung cancer, we had to use Steiger Test to test whether SNPs correlate with lung cancer more than ACE2, and set the threshold of *P* < 5 × 10^−5^ to exclude these SNPs strongly associated with lung cancer. By our statistical analysis, the extracted corresponding SNPs were not strongly associated with lung cancer. We assessed the horizontal pleiotropy of each IV in the PhenoScanner database (http://www.phenoscanner.medschl.cam.ac.uk/) and removed filtered IVs to reduce the impact of confounding variables. Further MR analysis was performed on the remaining IVs.

### Reverse MR analysis

**Figure 1:**
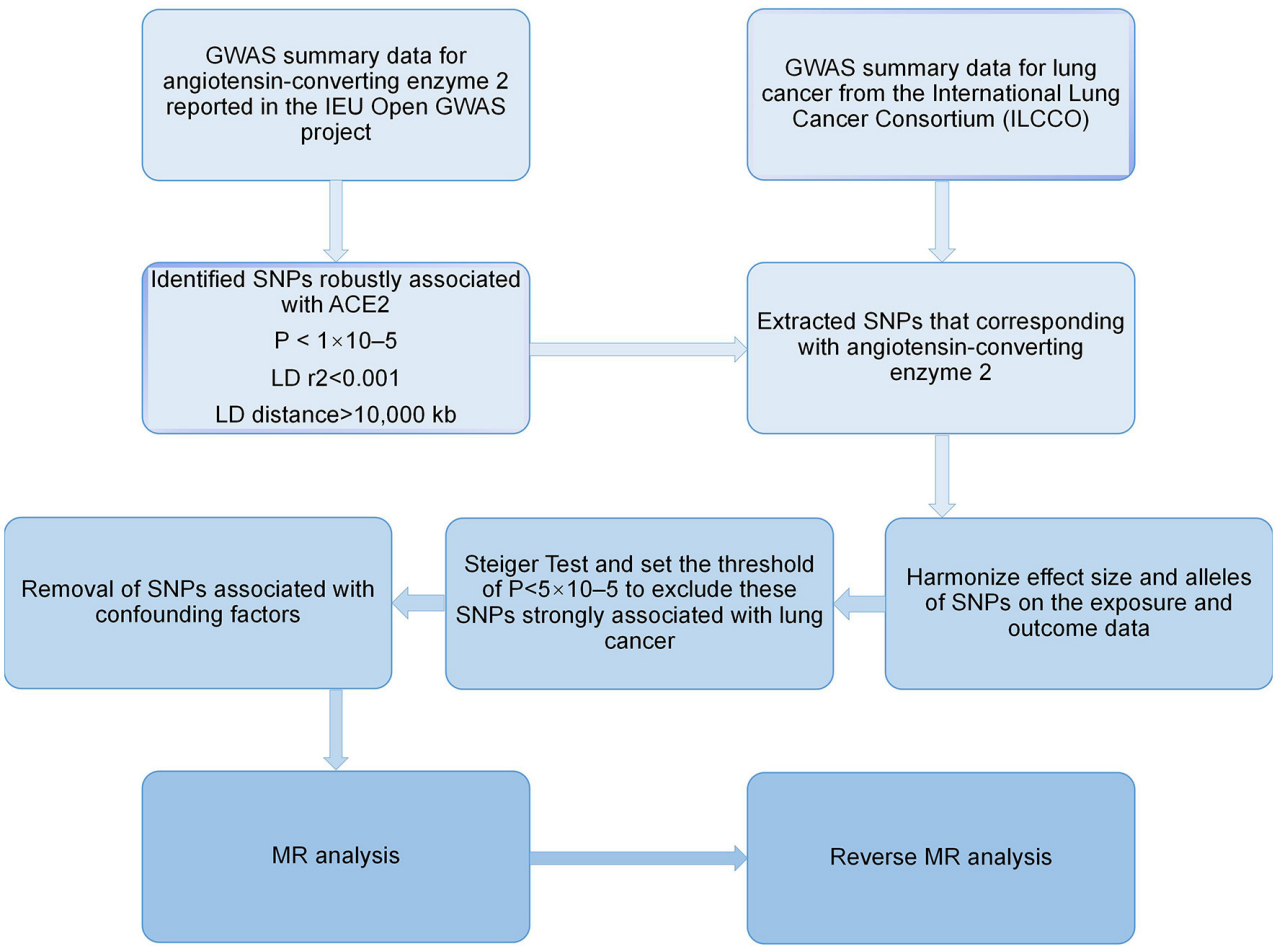
Flow chart of the Mendelian randomization study.

We also conducted a reverse MR analysis utilizing SNPs linked to lung cancer as IVs (lung cancer as the exposure and the ACE2 as the outcome) to investigate if lung cancer has any causal influence on the ACE2. Since the data for this study were made accessible to the public, informed consent and ethical review are not required. Figure 1 shows our study's layout.

### Statistical analysis

The connections between exposure (ACE2) and result (lung cancer) were determined using two-sample MR analysis. In order to calculate the impact of ACE2 on lung cancer, the inverse-variance weighted (IVW) technique is primarily utilized (28). The most accurate causal estimation is provided by this technique, which is comparable to a meta-analysis of the effects of a single SNP on lung cancer and is somewhat pleiotropy sensitive. Odds ratios (OR) and 95% confidence intervals (CI) were used to present the results. We evaluated causal relationship using MR Egger regression, weighted median (29), and weighted mode to account for the sensitivity of the data. Utilizing Cochran's Q statistics and the two-sample MR package across instruments, we tested for heterogeneity. Invalid instruments and heterogeneity can be demonstrated by a Q greater than the number of instruments minus one, or by Q statistics significant at a *p*-value of 0.05. The p-value of the MR-Egger regression intercept was utilized to evaluate the horizontal pleiotropy effect (29). There was a substantial pleiotropy bias when the *p* < 0.05. In order to track any potential horizontal pleiotropy impact, we additionally used MR-PRESSO testing. The MR-PRESSO global test generated a *p*-value for overall horizontal pleiotropy, whereas the MR-PRESSO outlier test calculated a *p*-value for each SNP's significance in terms of pleiotropy. *P*-value for the MR PRESSO global test is >0.05, indicating no horizontal pleiotropy. To further confirm the stability of the study, the leave-one-out analysis was carried out to determine whether the significant results were caused by a single SNP.

## Results

### Assessing ACE2 and the risk of lung cancer

**Figure 2:**
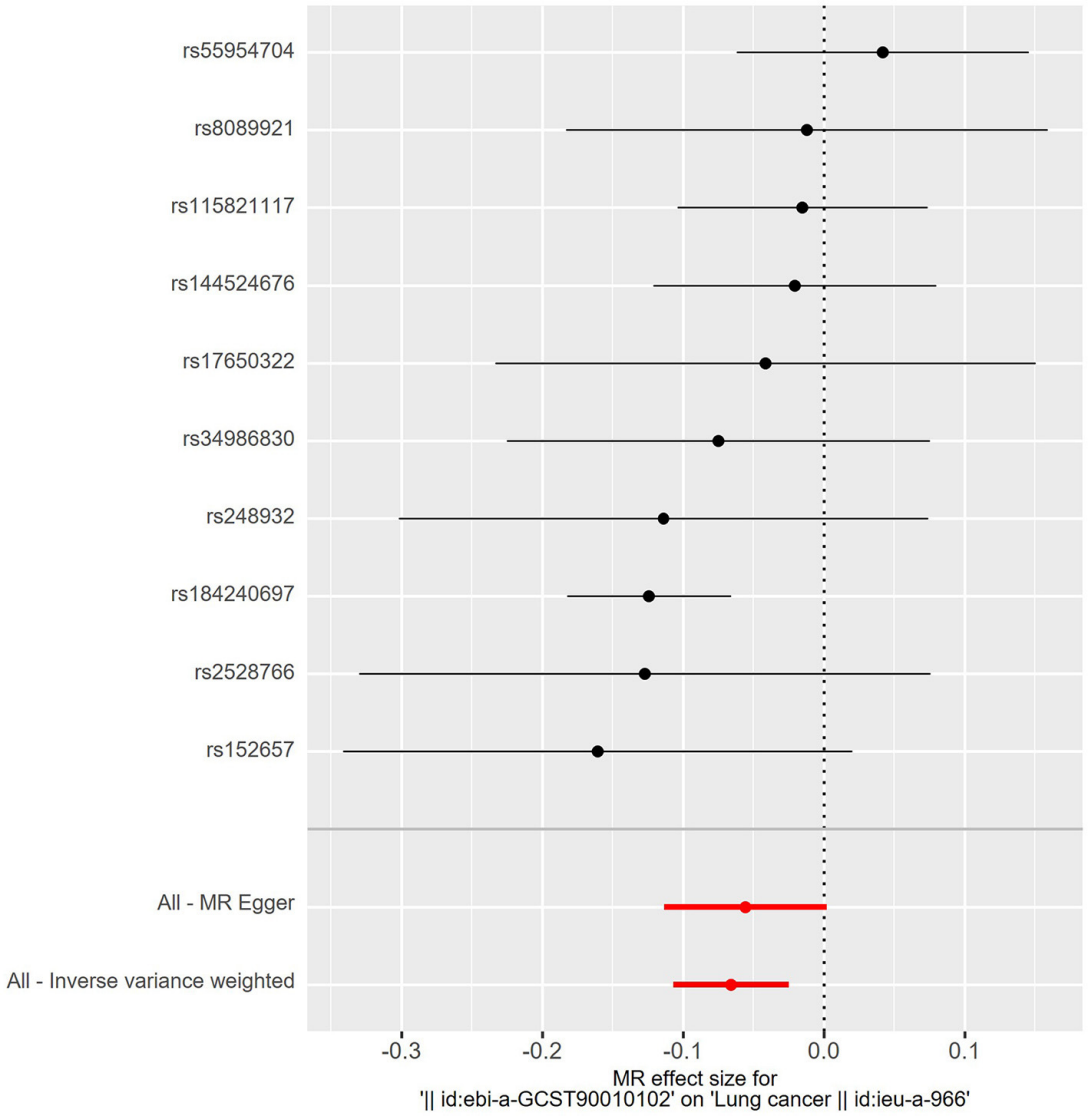
Forest plot of risk effects of each SNPs (rs55954704, rs8089921, rs115821117, rs144524676, rs17650322, rs34986830, rs248932, rs184240697, rs2528766, and rs152657) alone on lung cancer. Except for rs55954704, all other SNPs were protective factors. Statistically, higher ACE2 levels and a lower chance of developing lung cancer.

**Figure 3:**
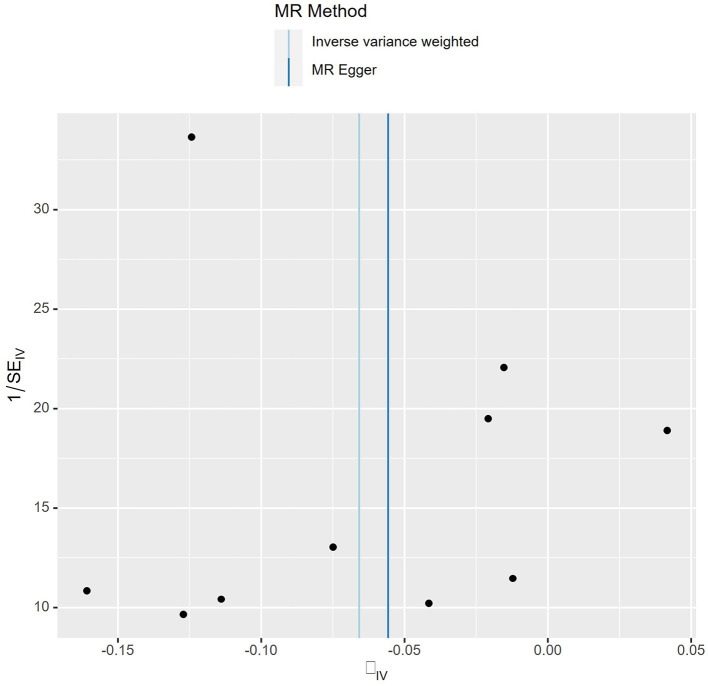
Funnel plot of MR analysis. Each point represents one SNP, and the overall left-right symmetry is pyramidal, indicating stable results and no significant bias in SNPs selection.

**Figure 4:**
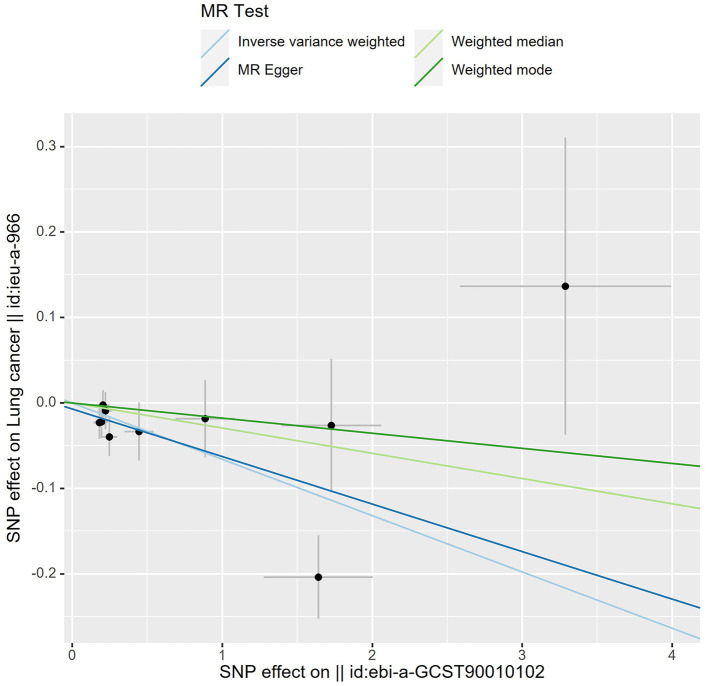
Scatter plot of MR analysis. A slope >0 is a positive correlation and < 0 is a negative correlation. Each black dot represents 1 SNP. The slope of lines for IVW and MR egger showed that higher ACE2 levels and a lower chance of developing lung cancer.

**Figure 5:**
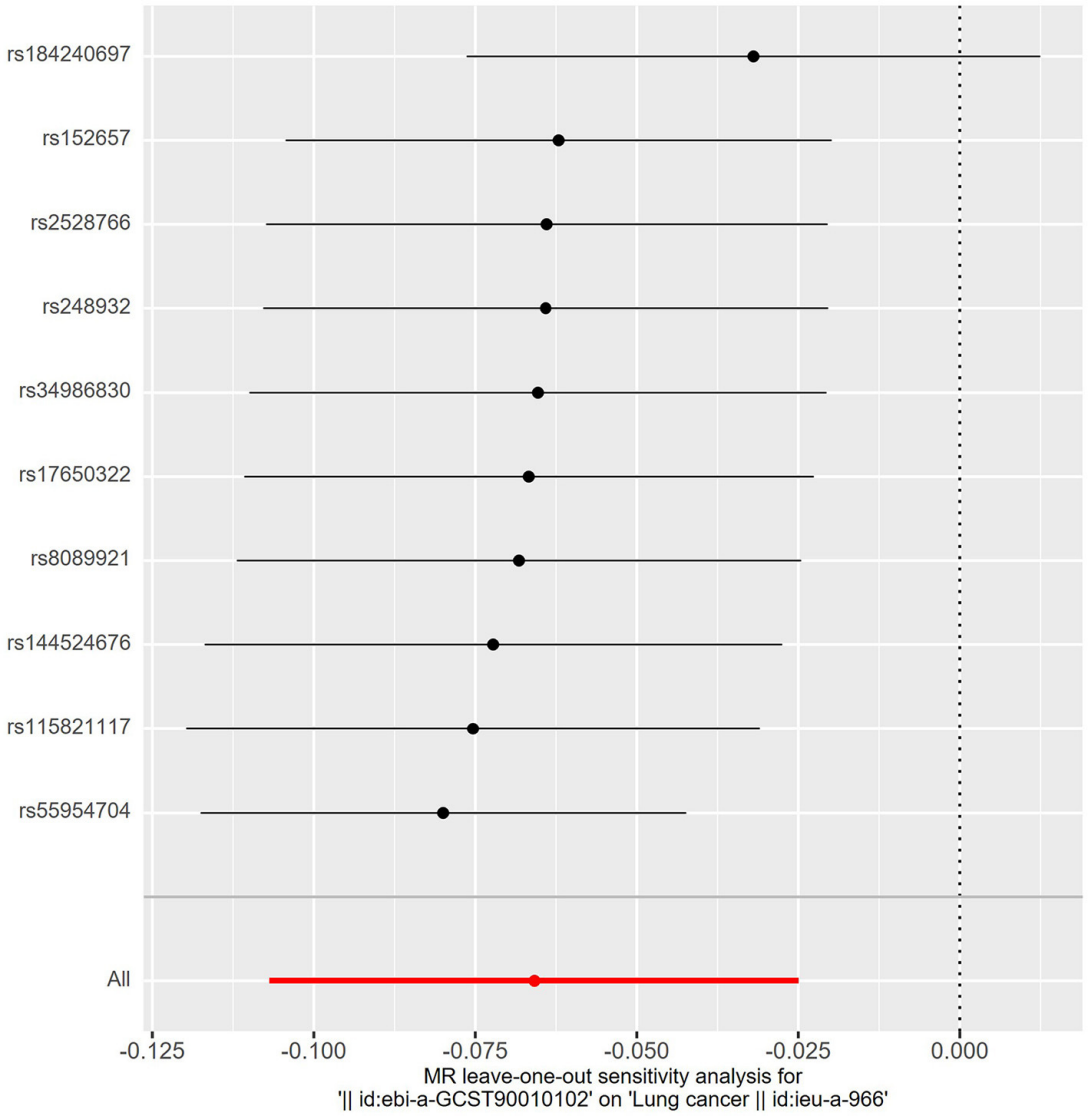
The leave-one-out analysis. Removing one SNP in turn and assessing the total effect of the remaining SNPs on risk of lung cancer, the results remained unchanged, indicating a stable result.

The details of SNPs associated to ACE2 were displayed in Table 1. Seven SNPs (rs12476354, rs1413219, rs3213545, rs6800514, rs72727274, rs75412100, and rs9652468) were linked to confounding factors (such as the monocyte count, hemoglobin concentration, total cholesterol, cardiovascular disease risk factors, and others) when we evaluated the horizontal pleiotropy of each IV in the PhenoScanner database. The findings of our MR analysis, which we conducted after deleting these confounding factors SNPs, indicated a substantial causation estimate between higher ACE2 levels and a lower chance of developing lung cancer (odds ratio: 0.94, 95% confidence interval: 0.90–0.98, *P* = 0.0016). Figure 2 depicts the effect of each SNP in detail. Cochrane's Q test showed that the IVW method, as the primary MR analysis, was not affected by heterogeneity in all analyses ($P_{\text{Cochran}{\text{e}}^{\text{′}}\text{s}}$_Q_ = 0.207) (Figure 3). Meanwhile, we found no evidence of horizontal pleiotropy in all analyses according to the MR-Egger intercept (P_intercept_ = 0.622). Finally, the MR-PRESSO global test did not recognize the existence of outlier SNPs (*P*_globaltest_ = 0.191) (Figure 4). To further confirm the stability of the study, the leave-one-out analysis was carried out to determine whether the significant results were caused by a single SNP. Removing one SNP in turn and assessing the total effect of the remaining SNPs on risk of lung cancer, the results remained unchanged, indicating a stable result (Figure 5). The specific results are displayed in Table 2.

**Table 1:** Summary information for 17 SNPs that were used as genetic instruments for Mendelian randomization analyses of ACE2.

**SNP**	**Effect allele**	**Other allele**	**Chr**	**EAF**	**Beta**	**SE**	***P***	**R^2^**	**F**
rs115821117	T	C	5	0.003	1.72661	0.332862	3.21E-07	0.017833	23.58625
rs12476354	A	G	2	0.165	0.253022	0.053345	2.95E-06	0.017641	23.32683
rs1413219	G	A	9	0.497	0.195949	0.040789	2.12E-06	0.019197	25.42541
rs144524676	A	G	5	0.011	0.885166	0.19788	9.90E-06	0.017048	22.52917
rs152657	C	T	16	0.168	0.246745	0.053728	5.65E-06	0.01702	22.49178
rs17650322	C	T	18	0.266	0.222245	0.045319	1.63E-06	0.019287	25.54695
rs184240697	T	C	14	0.003	1.64051	0.363503	8.47E-06	0.016099	21.25504
rs248932	C	A	19	0.332	−0.19439	0.043064	8.03E-06	0.01676	22.1426
rs2528766	G	A	13	0.476	−0.18129	0.040163	8.60E-06	0.016395	21.65208
rs3213545	A	G	12	0.317	−0.21928	0.043708	9.24E-07	0.020821	27.62098
rs34986830	G	C	6	0.046	−0.44486	0.097933	7.09E-06	0.017369	22.96147
rs55954704	C	T	1	0.001	3.28817	0.704338	3.96E-06	0.021602	28.68123
rs6800514	T	C	3	0.421	0.200619	0.039897	8.59E-07	0.019622	25.99861
rs72727274	G	A	1	0.018	0.769642	0.15316	8.75E-07	0.020941	27.7838
rs75412100	T	G	7	0.001	2.93387	0.575148	4.93E-07	0.017198	22.73109
rs8089921	T	C	18	0.346	0.203724	0.042814	2.65E-06	0.018783	24.86637
rs9652468	A	G	15	0.267	0.204507	0.045579	9.86E-06	0.01637	21.61918

**Table 2:** Two-sample MR analysis of ACE2 on the risk of lung cancer.

**Exposure**	**Outcome**	**Genetic instruments**	**MR-Egger intercept *P* value**	**Analysis method**	**OR (95% CI)**	***P* value**
ACE2	Lung cancer	17 SNPs before removing confounding factors	0.566	Inverse variance weighted	0.94 (0.92–0.97)	0.000293
				MR Egger	0.94 (0.90–0.98)	0.006954
				Weighted median	0.95 (0.91–0.99)	0.013608
				Weighted mode	0.94 (0.90–0.99)	0.023395
		10 SNPs after removing confounding factors	0.622	Inverse variance weighted	0.94 (0.90–0.98)	0.001616
				MR Egger	0.95 (0.90–1.00)	0.09547
				Weighted median	0.97 (0.91–1.03)	0.341679
				Weighted mode	0.98 (0.88–1.09)	0.748397

### Reverse-direction MR analysis to assess lung cancer and the risk of ACE2

We conducted a reverse-direction MR study to learn more about the connection between lung cancer and ACE2. The analysis followed the same format as before. Table 3 showed the specific characteristics of SNPs related to lung cancer. We assessed the horizontal pleiotropy of each IV in the PhenoScanner database and found that 12 SNPs (rs10866498, rs11710798, rs2075570, rs2647964, rs37004, rs446975, rs501942, rs66500423, rs75160488, rs76813064, rs79584940 and rs8040868) were associated with confounding factors (including whole body fat mass, mean corpuscular hemoglobin, body mass index and others). After removing these SNPs, we performed MR analysis and the results provided nonsignificant causal estimates between lung cancer and ACE2 (odds ratio: 0.94, 95% confidence interval: 0.68–1.30, *P* = 0.7209). The results of the *P* value of MR Egger regression, weighted median and weighted mode also did not show statistically significant. The specific results are displayed in Table 4.

**Table 3:** Summary information for 19 SNPs that were used as genetic instruments for Mendelian randomization analyses of lung cancer.

**SNP**	**Effect allele**	**Other allele**	**Chr**	**EAF**	**Beta**	**SE**	***P***	**R^2^**	**F**
rs10866498	T	C	5	0.475	−0.10316	0.01892	1.49E−06	0.005307	145.1606
rs11710798	C	A	3	0.118	−0.14173	0.025005	1.79E−06	0.004173	114.0148
rs13330749	A	T	16	0.229	−0.10716	0.019807	2.02E−06	0.004058	110.8486
rs147525635	A	G	15	0.549	0.092655	0.01798	4.17E−06	0.004251	116.1396
rs151606	A	T	6	0.660	−0.10939	0.02594	4.36E−06	0.005368	146.8415
rs2075570	T	C	1	0.499	0.090772	0.01644	7.45E−07	0.00412	112.55
rs2647964	G	A	12	0.455	0.088036	0.01971	1.69E−06	0.003843	104.9725
rs2816076	A	G	6	0.454	−0.08489	0.016551	3.75E−06	0.003573	97.56444
rs37004	T	C	5	0.226	−0.17209	0.019417	3.19E−13	0.010367	285.0042
rs446975	T	G	3	0.115	−0.26153	0.022649	6.42E−18	0.013937	384.5445
rs501942	T	C	6	0.109	0.186841	0.034137	1.47E−10	0.006773	185.5313
rs55791720	C	T	12	0.520	0.096108	0.016329	1.54E−07	0.004611	126.0304
rs66500423	C	T	19	0.318	0.096318	0.021238	9.82E−07	0.004022	109.862
rs73351721	A	G	12	0.058	0.204328	0.049704	1.32E−06	0.004597	125.6561
rs75160488	C	T	5	0.008	0.542572	0.178907	3.12E−06	0.00463	126.5496
rs76813064	C	T	3	0.062	0.197929	0.051011	5.91E−06	0.004531	123.8464
rs79097304	T	C	2	0.017	−0.32811	0.047593	3.60E−06	0.003657	99.85395
rs79584940	A	G	2	0.036	−0.23441	0.038747	5.40E−06	0.003855	105.2843
rs8040868	C	T	15	0.424	0.301974	0.024535	4.97E−60	0.044551	1268.617

**Table 4:** Two-sample MR analysis of lung cancer on the risk of ACE2.

**Exposure**	**Outcome**	**Genetic instruments**	**MR-Egger intercept *P* value**	**Analysis method**	**OR (95% CI)**	***P* value**
Lung caner	ACE2	19 SNPs before removing confounding factors	0.419	Inverse variance weighted	1.05 (0.90–1.22)	0.553853
				MR Egger	1.18 (0.85–1.65)	0.327347
				Weighted median	1.07 (0.85–1.35)	0.568655
				Weighted mode	1.07 (0.84–1.35)	0.599249
		7 SNPs after removing confounding factors	0.815	Inverse variance weighted	0.94 (0.68–1.30)	0.720864
				MR Egger	0.86 (0.38–1.96)	0.729059
				Weighted median	0.85 (0.56–1.29)	0.458728
				Weighted mode	0.80 (0.45–1.39)	0.453421

## Discussion

The relationship between ACE2 and the risk of developing lung cancer was described in this two-sample MR investigation. Using IEU Open GWAS data sets, we showed a clear causal relationship between ACE2 and a decreased risk of developing lung cancer in this study. In addition, a reverse-direction MR examination revealed no conclusive evidence of a causal relationship between lung cancer and ACE2. As far as we are aware, this is the first MR research to look into the connections between ACE2 and risk of lung cancer.

A link between ACE and the risk of lung cancer has previously been hypothesized by observational research. According to Romer (30), who first established the link between ACE and lung cancer risk in 1981, low ACE is linked to a poor prognosis for those with lung cancer. A clinical trial carried out by Bar and other researchers (31) revealed that lung cancer patients with high baseline ACE levels had much better outcomes than those with low ACE levels. ACE was a preventive factor against lung cancer, as shown by all of the aforementioned investigations. To be fair, ACE2 shares structural similarities with ACE and performs biologically in a manner that is not dissimilar from that of ACE, however, there was few research that discussed the connection between ACE2 and lung cancer. Li et al. reported on the association between ACE2 and the risk of major pulmonary resection in non-small cell lung cancer (32). Their findings revealed that patients with a serum ACE2 level < 3.21 ng/mL had significantly higher rates of pneumonia, pleural effusion, and atrial fibrillation as well as higher in-hospital mortality following major pulmonary resection than those with a serum ACE2 level > 3.21 ng/mL. Inhibiting cell growth and VEGFa production *in vitro*, the work by Feng et al. (20) shown that overexpression of ACE2 may have a protective impact. According to the research stated above, ACE2 may be a lung cancer preventative. Yamaguchi (33) presented the findings of research that demonstrated that the amount of ACE2 expression in cancer cells was much greater than that in healthy lung epithelial cells, in contradiction to several studies that claimed ACE2 is a risk factor for lung cancer. In the same way, Samad et al. (34) argued in a 2020 paper that increased expression of ACE2 was linked to a bad prognosis for lung cancer. The association between ACE2 and the risk of lung cancer had been contentious since all types of retrospective and experimental investigations were susceptible to confounding variables that might distort results. Our research demonstrated the causal relationship between ACE2 and a decreased risk of developing lung cancer in an effort to validate MR assumptions.

There are several logical possibilities, even if the exact mechanism by which higher ACE2 levels were linked to a decreased chance of developing lung cancer is yet unknown. It indicated that the equilibrium of circulating Ang II/Ang 1-7 levels was controlled by pulmonary ACE2. In patients with lung damage, Ang II causes pulmonary vasoconstriction in response to hypoxia, which is crucial in avoiding shunting. It indirectly implies that the high expression of ACE2 might result in a lower risk of developing lung cancer since lung damage is frequently one of the prerequisites for the development of lung cancer (35). According to the research by Feng et al. (20), overexpressing ACE2 caused lung cancer cells to proliferate at a significantly higher rate in the G0/G1 phase than they did in the G2/M phase. Meanwhile, lung cancer cells can produce less VEGFa protein and accumulate less VEGFa mRNA when ACE2 is overexpressed. When compared to the vector, gene expression investigation by (qRT-PCR) revealed that the VEGFa mRNA level was lower in lung cancer cells infected with MSCV-ACE2. It has also been extensively shown in a different study (36) that ACE2 overexpression suppresses tumor angiogenesis in NSCLC. Cell proliferation and angiogenesis are linked to tumor formation, and ACE2 further inhibits these processes, allowing killer immune cells more time to identify and eradicate tumor cells. The mechanism known as epithelial-mesenchymal transition (EMT) is increasingly recognized as being crucial to the development and spread of tumors. The research by Qian et al. (21) revealed a correlation between ACE2 overexpression and lower mRNA levels of genes that are causally connected to the EMT process, including Snail2, ZEB1, and Twist. A traditional EMT model was created utilizing lung cancer cells treated with TGF-1 in order to further examine whether ACE2 reduces the EMT procedure. Lung cancer cells that contained ACE2 slowed the decline in E-cadherin levels brought on by TGF-1 therapy. The researches mentioned above have shown that ACE2 has a preventative impact on the growth of lung cancer, both theoretically and *in vitro*.

Cardiovascular disorders are commonly treated with angiotensin-converting enzyme inhibitors (ACEIs) and angiotensin receptor blockers (ARBs). The majority of research currently demonstrates that long-term ACEI usage raises the risk of lung cancer (10–12). However, there is still a lot of disagreement regarding the connection between ACEI/ARB administration and ACE2 expression. Consistent findings from studies utilizing spontaneously hypertensive rats showed that ACEI or ARB therapy elevated ACE2 expression (37–40). However, the cardiac and renal systems received most of these research' attention, and the impact on ACE2 expression in the lung was not further explored. The findings of a research by Han et al. (41) examining Losartan's effects on ACE2 expression in the lung were in agreement with the studies mentioned above in that ARBs raised ACE2 expression in the lung. It may be said that evidence for the elevation of ACE2 in response to ACEI or ARB therapy was presented in two-thirds of the animal investigations. The following information may be used to explain how ACE2 is upregulated in response to ACEI/ARB: When Ang II acts on AT1 receptors, it causes ACE2 to internalize, Ang II decreases ACE2 expression by activating the ERK1/2 or p38 MAPK pathways, and RAS inhibition by ACEI/ARB leads in higher tissue levels of ACE2 (42, 43). We should do extensive clinical and fundamental research to show how ACEI/ARB affect ACE2 expression in the lung in light of the aforementioned findings. This implicitly implies that the role of the RAS in the formation of lung cancer is complicated and that ACEI/ARB medication may have an impact on lung cancer development via additional pathways, such as the buildup of substance P in the lungs due to ACEI usage. Substance P could encourage angiogenesis by causing endothelial and tumor cell proliferation (44). One mechanism connecting mitogenesis with the growth and progression of cancer is the activation of neurokinin-1 receptors by substance P, according to another study (45). If the aforementioned validation is accurate, future researchers might focus on ACEI/ARBs for more modification to significantly enhance the quantity of ACE2 produced while it functions, further mitigating the harm caused by substance P.

This study's main advantage is using data from genetic studies and large consortiums to assess the relationship between ACE2 and lung cancer, thus largely avoiding the common bias of observational studies. Secondly, to investigate the relationship between AEC2 and lung cancer, we rigorously implemented the three key hypotheses of MR and designed a statistically rigorous analysis. Public health initiatives focused on lowering the risk of lung cancer may be affected by these findings and these findings could guide drug scientists in the direction of further ACEI/ARB drug research in the future.

It is important to think about some potential study limitations. The GWAS employed in this work was based on the European population, therefore additional research is needed to see whether our findings can be generalized to other groups. Second, the association between ACE2 and lung cancer was only briefly examined in our study; it did not examine the relationship between ACE2 and certain forms of lung cancer, such as lung adenocarcinoma, squamous lung cancer, or small cell lung cancer. Last but not least, despite the fact that we employ a variety of techniques to mitigate the effects of pleiotropy, we are unable to totally rule out the bias that unknown pleiotropy may have on the outcomes. The link between ACE2 and lung cancer must thus be clarified by more large-scale prospective clinical trials, fundamental science research, and GWAS data from ethnically varied populations.

## Conclusion

In conclusion, an important causal relationship between ACE2 and the likelihood of developing lung cancer was found by our MR study. Public health initiatives focused at lowering the risk of lung cancer may be affected by these findings.

## Data availability statement

The original contributions presented in the study are included in the article, further inquiries can be directed to the corresponding authors.

## Ethics statement

This study was based on publicly available datasets. Ethical review and approval was not required for the study, in accordance with the local legislation and institutional requirements. Written informed consent for participation was not required from the participants or the participants' legal guardians/next of kin in accordance with the national legislation and institutional requirements because since the data for this study were made accessible to the public.

## Author contributions

SC: Writing – original draft. RN: Writing – review & editing. WJ: Data curation, Writing – review & editing. SZ: Methodology, Writing – review & editing. QY: Conceptualization, Writing – review & editing. HG: Supervision, Writing – review & editing.
